# Beyond NAD Depletion: SARM1-Induced ATP Collapse Involves Direct ATP Degradation and Mitochondrial Dysfunction and Is Pharmacologically Reversible

**DOI:** 10.3390/biology15171552

**Published:** 2026-09-04

**Authors:** Zhuo Chen, Zhi Ying Zhao, Zi-Mei Zhang, Ruoyu Zhao, Linru Shen, Hon Cheung Lee, Lei Fang, Yong Juan Zhao

**Affiliations:** 1 Division of Biomedical Health Sciences, School of Medicine, The Chinese University of Hong Kong, Shenzhen 518172, China; 222050014@link.cuhk.edu.cn (Z.C.); zhangzimei@cuhk.edu.cn (Z.-M.Z.);; 2 State Key Laboratory of Chemical Oncogenomics, Key Laboratory of Chemical Genomics, Peking University Shenzhen Graduate School, Shenzhen 518055, China; zzypku2020@163.com; 3 Jiangsu Key Laboratory of Molecular Medicine, Chemistry and Biomedicine Innovation Center, Medical School, Nanjing University, Nanjing 210093, China

**Keywords:** SARM1, sarmoptosis, ATP collapse, ATP consumption, VER-155008, CZ-48, mitochondrial dysfunction, iTRAQ

## Abstract

Sterile alpha and Toll/interleukin-1 receptor motif-containing protein 1 (SARM1) is an inducible NAD-consuming enzyme and execution factor in axon degeneration. Rapid ATP collapse after SARM1 activation, however, is not fully explained by NAD depletion alone. We used SARM1-overexpressing HEK293 cells and the cell-permeant activator CZ-48 to examine SARM1-induced non-apoptotic cell death, termed sarmoptosis. CZ-48 induced cell death that was suppressed by HSP90/70-annotated ATP-competitive compounds, especially geldanamycin and VER-155008 (VER), without reducing SARM1 abundance. VER preserved NAD and ATP during SARM1 activation but failed to rescue FK866-mediated NAD starvation, thereby distinguishing CZ-48/SARM1-driven cytotoxicity from generic NAD depletion. In cell-free assays, purified SARM1 reduced ATP levels; this effect was enhanced by SARM1’s activator NMN and attenuated by its pharmacological inhibitors, although the *in vitro* activity was modest and the reaction products remain to be identified. ATPase-related perturbations, including thapsigargin and bafilomycin A1, also protected cells from CZ-48-induced death, further supporting a central role for ATP collapse in sarmoptosis. iTRAQ proteomics, MitoSOX Red staining, and DiOC6(3) staining revealed that CZ-48 treatment was associated with mitochondrial and metabolic remodeling, mitochondrial ROS accumulation, and mitochondrial depolarization, all of which were mitigated by VER. Collectively, these findings support a convergent ATP-collapse model in which SARM1 activation promotes NAD depletion, directly consumes ATP, and is associated with mitochondrial dysfunction that may amplify ATP-production failure.

## Simple Summary

SARM1 is a protein that plays an important role in the degeneration of injured nerve fibers. When SARM1 is activated, cells rapidly lose ATP, the main molecule that supplies cellular energy. However, this rapid energy loss cannot be fully explained by depletion of NAD, another key molecule required for cell metabolism. In this study, we used HEK293 cells engineered to overexpress SARM1 and activated SARM1 with the cell-permeable compound CZ-48. We found that CZ-48 caused a regulated, non-apoptotic form of cell death, referred to as sarmoptosis. Several compounds, especially VER-155008, protected cells from this death without reducing SARM1 protein levels. VER-155008 helped maintain both NAD and ATP during SARM1 activation, but it did not protect cells from NAD depletion caused by a different mechanism, suggesting that SARM1-induced death involves more than general NAD loss. We also found evidence that SARM1 may directly reduce ATP levels and that mitochondrial dysfunction contributes to this process. Overall, our findings suggest that SARM1-induced cell death is driven not by NAD depletion alone, but by multiple converging mechanisms that collectively trigger ATP collapse.

## 1. Introduction

Regulated cell death proceeds through several biochemical programs, not through a simple split between apoptosis and accidental necrosis. The Nomenclature Committee on Cell Death recognizes apoptosis, necroptosis, ferroptosis, pyroptosis, parthanatos, and related forms of regulated necrosis [1,2]. Necroptosis is one of the better-defined necrosis-like pathways and is usually driven by receptor-interacting protein kinase 1 (RIPK1), RIPK3, and mixed lineage kinase domain-like protein (MLKL), ending in MLKL-dependent membrane disruption [3]. However, plasma membrane rupture, propidium iodide (PI) uptake, cell swelling or shrinkage, and blebbing can also appear in other death programs. Morphology alone therefore cannot assign a dying cell to canonical necroptosis. Enzyme-driven metabolic collapse may produce necrotic phenotypes that overlap with, but remain mechanistically distinct from, RIPK1-RIPK3-MLKL signaling.

SARM1 is one such death-associated enzyme. The SARM1-dependent, non-apoptotic cell death pathway was first termed “sarmoptosis” in 2014, when SARM1 was shown to drive axon degeneration and neuronal death downstream of mitochondrial dysfunction and oxidative stress in sensory neurons [4]. Genetic studies then placed SARM1 as an essential mediator of injury-induced axon degeneration, with loss of dSarm/Sarm1 protecting injured axons in *Drosophila* and mice [5]. Later work showed that SARM1 activation is sufficient to trigger local axonal NAD depletion and degeneration [6]. The biochemical basis became clear when the SARM1 Toll/interleukin-1 receptor (TIR) domain was shown to have intrinsic NAD cleavage activity [7] and to be activated by nicotinamide mononucleotide (NMN) [8]. Activated SARM1 hydrolyzes NAD and can generate cyclic ADP-ribose (cADPR) and related products, revealing that a domain class traditionally regarded as a protein–protein interaction adaptor module in innate immune signaling can also function as a metabolic execution module [8,9,10,11,12].

Although SARM1 has been studied mainly in axon degeneration, its activation also induces cell death including in neurons [13] and T cells [14]. We previously established a reductionist system in SARM1-overexpressing HEK293 cells, where the cell-permeant NMN mimetic CZ-48 activates SARM1, increases cADPR production and NAD depletion, and induces non-apoptotic cell death [8]. This model removes the anatomical complexity of axons while retaining core metabolic features of SARM1-dependent degeneration. It provides a tractable way to ask how SARM1 activation becomes terminal cellular failure.

A major unresolved issue is how SARM1 activation produces rapid ATP collapse. SARM1-mediated NAD degradation should impair ATP synthesis by limiting NAD-dependent reactions in glycolysis, the tricarboxylic acid cycle, and mitochondrial oxidative phosphorylation. Yet NAD depletion alone may not explain the speed and severity of ATP loss after SARM1 activation. The cellular ATP pool depends on ATP production, degradation, and consumption. ATP collapse during sarmoptosis may therefore reflect impaired synthesis, enhanced ATP breakdown, persistent ATP-consuming activity, or a combination of these processes.

Activated SARM1 itself may contribute directly to ATP loss. SARM1 contains a catalytically active TIR domain, and TIR domains are now recognized as nucleotide-metabolizing modules rather than passive signaling domains [15,16]. In plants, TIR-domain enzymes catalyze ADP-ribosylation reactions that use ATP or ADP as nucleotide substrates or acceptors to generate pRib-AMP/ADP immune signaling molecules [17,18]. These findings raised the possibility that activated SARM1 has ATP-metabolizing activity in addition to its established NADase activity. At the same time, constitutive ATP-consuming processes, including ion transport, organellar acidification, protein folding, and vesicular trafficking, may accelerate ATP exhaustion when SARM1-mediated NAD loss compromises ATP production. ATP synthesis may also be limited by mitochondrial dysfunction, including impaired membrane potential, redox imbalance, and defective oxidative phosphorylation [19].

Pharmacology provided an entry point into this mechanism. We identified several compounds that protect cells from CZ-48-induced death, including geldanamycin and compounds originally selected or annotated as HSP70/HSP90-related inhibitors [20,21]. Many of these molecules are ATP-competitive or ATPase-sensitive compounds. Their protection may therefore reflect interference with SARM1-linked nucleotide metabolism, ATP-consuming processes, or mitochondrial energy stress.

Here, we used CZ-48-treated SARM1-overexpressing HEK293 cells to examine how SARM1 activation drives ATP collapse and non-apoptotic death. We compared CZ-48-induced SARM1 activation with FK866-mediated NAMPT inhibition to separate SARM1-specific ATP loss from generic NAD starvation. We then tested whether activated SARM1 can directly consume ATP, whether reducing selected ATP-consuming processes alters cell death, and whether mitochondrial dysfunction contributes to ATP failure. iTRAQ proteomics and mitochondrial staining were used to map broader ATP-collapse-associated pathways. These data support a pharmacologically reversible, multi-route model in which SARM1 activation drives sarmoptosis through NAD depletion, direct ATP consumption, and probably mitochondrial dysfunction.

## 2. Materials and Methods

### 2.1. Cell Culture and SARM1 Activation

SARM1-overexpressing HEK293 cells (ATCC, CRL-1573, Manassas, VA, USA) were constructed with lentivirus infection based on pCDH-SARM1 [8] and maintained in Dulbecco’s modified Eagle’s medium supplemented with 10% fetal bovine serum and 1% penicillin–streptomycin at 37 °C in a humidified 5% CO_2_ incubator.

The information on plasmids, antibodies, and chemical reagents is in Supplementary Table S1.

### 2.2. Propidium Iodide (PI) Staining

Cell death was quantified by PI staining. After treatment, cells were harvested, washed with ice-cold phosphate-buffered saline, stained with 1 μg/mL PI at room temperature for 20 min, and analyzed by flow cytometry (CytoFlex, Beckman Coulter, Brea, CA, USA; ex/em: 488 nm/550 nm). PI-positive cells were quantified as a percentage of total analyzed cells.

### 2.3. Immunoblotting

Cells were lysed in ice-cold lysis buffer (50 mM Tris (pH 8.0), 150 mM NaCl, 1 mM EDTA, 0.5% TritonX-100) supplemented with protease inhibitors. Protein concentrations were measured using Bradford. Equal amounts of protein were separated by SDS-PAGE, transferred to PVDF membranes, and probed with antibodies against SARM1 and tubulin. Tubulin was used as a loading control. Signals were developed using ECL (Vazyme, Nanjing, China) and detected with a Chemidoc MP system and ImageLab v6.1 software (BIO-RAD, Hercules, CA, USA).

### 2.4. Preparation and SDS-PAGE Analysis of Recombinant SARM1

Recombinant SARM1 was purified from dt-SARM1 overexpression Expi293F cells, which were constructed with lentivirus infection based on pLenti-dtSARM1 [10]. Briefly, cells were expanded and harvested, and the cytosolic fraction was extracted with ice-cold PBS containing 200 μM digitonin and protease inhibitors. The cleared lysate was incubated with StrepTactin™ (IBA Lifesciences GmbH, Göttingen, Germany) resin overnight at 4 °C, washed with the buffer containing 100 mM Tris, pH 8.0, 150 mM NaCl, and 3 mM EDTA, and eluted with 2 mM biotin. The eluate was concentrated using a 100-kDa Amicon filter and further purified by size-exclusion chromatography on a Superdex 200 Increase 10/300 GL column (Cytiva, Uppsala, Sweden). The purified recombinant SARM1 was analyzed by SDS-PAGE followed by Coomassie Brilliant Blue staining. The gel was then destained and imaged (BIO-RAD, ChemiDoc, Hercules, CA, USA).

### 2.5. Cellular NAD and ATP Measurements

For nucleotide measurements, treated cells were resuspended in 0.6 M perchloric acid and centrifuged at 13,000 rpm for 10 min at 4 °C. The supernatant was collected, and the pellet was dissolved in 1 M NaOH for protein quantification. The perchloric acid-containing supernatant was mixed with an organic solvent mixture consisting of tri-n-octylamine and chloroform (1:3, *v*/*v*), followed by centrifugation at 13,000 rpm for 10 min at 4 °C. An aliquot of the resulting aqueous phase was diluted for NAD and ATP measurements. NAD was determined using enzymatic cycling assays adapted from established methods [22]. Briefly, samples were incubated with a reaction mixture containing 2% ethanol, 100 μg/mL alcohol dehydrogenase, 10 μM resazurin, 5 μg/mL charcoal-treated diaphorase, and 10 μM FMN in PBS. Fluorescence was measured in white 96-well plates (Corning, Corning, NY, USA) using the SPARK microplate reader (Tecan, Männedorf, Switzerland) at an excitation wavelength of 544 nm and an emission wavelength of 576 nm. NAD concentrations were determined from a standard curve generated using known concentrations of NAD. ATP levels were measured using a luminescence-based ATP detection kit (Beyotime, Shanghai, China) according to the manufacturer’s instructions. Briefly, each diluted sample was mixed with ATP detection reagent and incubated at room temperature for 10 min. Luminescence was measured using an EnVision 2105 Multimode Plate Reader (PerkinElmer, Waltham, MA, USA).

### 2.6. Cell-Free ATP-Consumption Assay

SARM1-associated ATP consumption was tested using the cell-free assay shown in Section 3.3. Briefly, purified recombinant SARM1 [10] was incubated with ATP under the indicated activation or inhibitory conditions for 5 h, unless otherwise specified. The standard reaction mixture contained 2 μM SARM1, 0.18 μM ATP, and 1 mM Mg^2+^ in PBS. Where indicated, NMN was added to activate SARM1, and Zn^2+^, dHNN, or VER was added to evaluate their effects on SARM1-associated ATP consumption. Control reactions contained ATP alone or ATP incubated with BSA instead of SARM1. After incubation, ATP levels were measured using a luminescence-based ATP detection kit, as described in Section 2.5, and luminescence was measured using an EnVision 2105 Multimode Plate Reader (PerkinElmer, Waltham, MA, USA). ATP consumption rates were calculated from the decrease in ATP concentration over time and expressed as nM ATP/h; data analysis and graph generation were performed using GraphPad Prism 10.4.0.

### 2.7. PC6 Assay for SARM1 NAD-Related Activity

SARM1 NAD-base-exchange activity was measured *in vitro* using a fluorescent PC6 assay as previously described [23]. Briefly, the standard reaction mixture contained 0.13 μM recombinant SARM1, 50 μM PC6, and 100 μM NAD in PBS. Where indicated, NMN, Zn^2+^, dHNN, or VER-155008 was added to activate or inhibit SARM1. Fluorescence of the product PAD6 was measured in black 96-well plates using a SPARK microplate reader (Tecan, Männedorf, Switzerland) with excitation at 390 nm and emission at 520 nm. The initial reaction rate was calculated from the slope of the fluorescence increase during the initial linear phase and expressed as relative fluorescence units per minute (RFU/min). Data analysis and graph generation were performed using GraphPad Prism 10.4.0.

### 2.8. iTRAQ-Based Quantitative Proteomics

Cells were seeded in six-well plates at 8 × 10^5^ cells per well and treated with vehicle, 100 μM CZ-48, 20 μM VER, or both compounds for 16 h. These groups are referred to as Ctrl, CZ, VER, and CZ+VER, respectively, for simplicity. After treatment, cells were washed with cold PBS, collected, and lysed in buffer containing 50 mM Tris-HCl, pH 8.0, 150 mM NaCl, 1 mM EDTA, 0.5% Triton X-100, and protease inhibitors. Lysates were incubated on ice for 25 min and centrifuged at 13,000 rpm for 10 min at 4 °C. Protein concentrations were determined by Bradford assay. For each sample, 45 μg protein was analyzed by SDS-PAGE and Coomassie Brilliant Blue staining to assess sample quality, and the remaining protein was used for iTRAQ analysis.

Proteins were reduced, alkylated, digested with trypsin, and labeled using an iTRAQ 8Plex multiplex reagent kit [24,25] according to the manufacturer’s protocol. Labeled peptides were pooled, fractionated, desalted, concentrated, and analyzed by LC-MS/MS (TripleTOF 5600+, SCIEX, Marlborough, MA, USA). Raw spectra were searched against the *Homo sapiens* UniProt database, with the SARM1 construct sequence included when necessary. Trypsin was set as the digestion enzyme, iTRAQ labeling and cysteine alkylation as fixed modifications, and methionine oxidation as a variable modification. Protein identification was controlled using a target-decoy strategy with a false discovery rate below 1%.

The normalized protein expression data are shown in Supplementary Table S2.

### 2.9. Bioinformatic Analysis

All bioinformatic analyses were performed using R software (version 4.4.1). iTRAQ protein abundance ratios were treated as relative quantitative measurements, normalized to the reference sample and log_2_-transformed before downstream analysis. Principal component analysis was used to assess global proteomic variation among experimental groups. Pairwise differential-abundance analyses were performed using the limma framework, and proteins satisfying *p* < 0.05 and |FC| ≥ 1.2 were considered differentially abundant. Volcano plots were used to summarize pairwise proteomic changes, whereas hierarchical clustering heatmaps were generated from row-wise Z-score-normalized abundance matrices. The HSP70 client protein set was derived from the proteome-wide HSP70/HSC70 chaperone-client study by Ryu et al. [26], in which HSP70-binding proteins were identified using the ubiquitin-activated interaction trap (UBAIT) coupled with quantitative mass spectrometry in human cells. The original study reported 214 proteins shared across three independent HSP70-UBAIT experiments; re-analysis of the deposited Supplementary Table S2 identified 213 reproducible non-bait proteins classified as enriched in all three experiments, which were used as the HSP70 client protein set in the present study (Supplementary Table S3, column 2).

For the analysis of chaperone or mitochondrial association, two predefined protein sets were curated: mitochondrial proteins and the client proteins of chaperones. The mitochondrial protein set was obtained from the Human Protein Atlas and comprised 1116 proteins annotated as mitochondrial (Supplementary Table S3, column 1).

For the mechanism-focused analysis, CZ-48-responsive, VER-opposed proteins were defined more stringently as proteins that simultaneously satisfied *p* < 0.05 and |FC| ≥ 1.2 in both the CZ-versus-Ctrl and CZ-versus-CZ+VER comparisons and whose mean abundance in the CZ-48-treated group was either higher than in both the Ctrl and CZ+VER groups or lower than in both groups. This bidirectional definition therefore identified both proteins induced by CZ-48 and proteins suppressed by CZ-48 whose abundance changes were opposed following VER co-treatment, thereby prioritizing candidates potentially associated with CZ-48-induced cellular injury and its modulation by VER.

The focused heatmaps in Section 3.4 were generated using different selection criteria. The first heatmap included iTRAQ-quantified proteins belonging to the predefined HSP70 client protein set. The second heatmap included the 65 CZ-48-responsive, VER-opposed proteins identified using the stringent bidirectional criteria, as described above, without restriction to a predefined functional protein set. The third heatmap included iTRAQ-quantified mitochondrial proteins belonging to the predefined mitochondrial protein set. Representative proteins in the third heatmap were selected to cover major mitochondrial processes, including oxidative phosphorylation, mitochondrial translation, redox metabolism, mitochondrial chaperone activity, and related metabolic pathways. Thus, the third heatmap was used to summarize mitochondrial proteomic remodeling rather than to display canonical or highly specific mitochondrial markers. These focused panels were used as mechanism-guiding summaries of the proteomic data.

### 2.10. Mitochondrial Reactive Oxygen Species and Membrane Potential

Mitochondrial reactive oxygen species (ROS) and membrane potential were measured using MitoSOX Red [27] or DiOC6(3) staining [28] methods. After the indicated treatments, live cells were incubated with 5 µM MitoSOX Red for 15 min or 40 nM DiOC6(3) for 30 min at 37 °C, respectively. The cells were then washed with PBS and analyzed by flow cytometry (CytoFlex, Beckman Coulter, Brea, CA, USA; ex/em: 488 nm/580 nm or 482 nm/504 nm, respectively). Fluorescence intensity or gated positive populations were quantified from biological replicates [8].

### 2.11. Statistics and Reproducibility

Statistical tests were performed using GraphPad Prism 10.4.0. Unless otherwise stated, data were obtained from at least three independent experiments, each including three technical replicates per condition; the iTRAQ experiment was performed with two biological replicates per condition. For multi-group comparisons, one-way analysis of variance (ANOVA) was employed, while unpaired Student’s *t*-tests were used for two-group comparisons, as specified in the figure legends. All statistical tests were two-tailed, with a *p*-value threshold of less than 0.05 deemed significant. The significance levels are denoted as follows: ns, not significant; *, *p* < 0.05; **, *p* < 0.01; ***, *p* < 0.001; ****, *p* < 0.0001.

## 3. Results

### 3.1. CZ-48-Induced SARM1 Activation Causes Cell Death That Is Suppressed by Geldanamycin but Is Not Fully Consistent with Canonical Necroptosis

**Figure 1:**
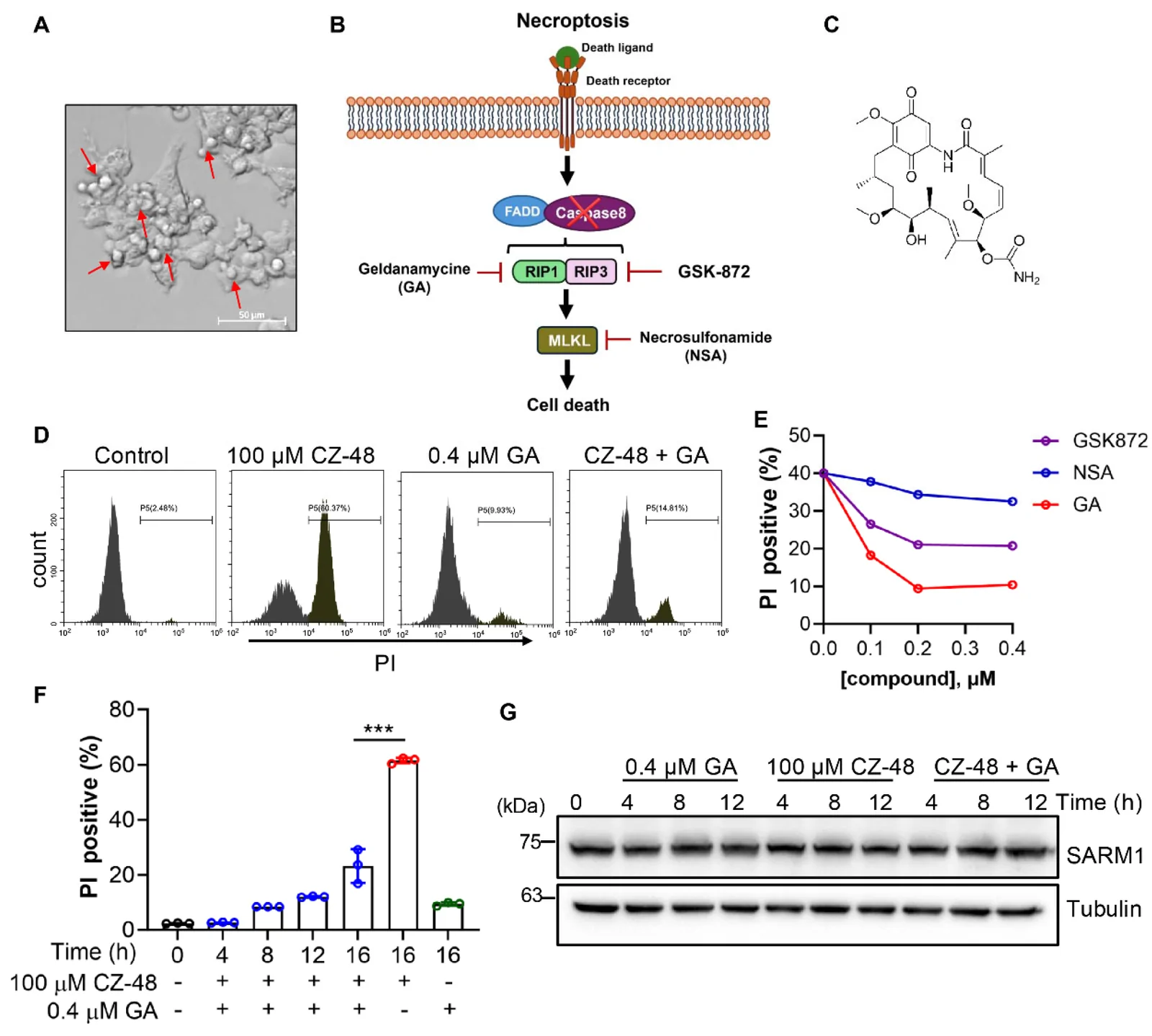
Geldanamycin suppresses CZ-48-induced cell death in SARM1-overexpressing HEK293 cells. (**A**) Representative phase-contrast image of SARM1-overexpressing HEK293 cells treated with 100 μM CZ-48 for 16 h. Red arrows mark blister-like cells. Scale bar, 50 μm. (**B**) Schematic of the canonical necroptosis pathway and the tested intervention points for geldanamycin (GA), GSK872, and necrosulfonamide (NSA). (**C**) Chemical structure of GA. (**D**) Representative flow-cytometry histograms of PI staining in cells treated with vehicle, 100 μM CZ-48, 0.4 μM GA, or CZ-48 plus GA for 16 h. The gate indicates the PI-positive population. (**E**) Quantification of PI-positive cells after 100 μM CZ-48 treatment with increasing concentrations of GA, NSA, or GSK872 for 16 h. (**F**) Time-course quantification of PI-positive cells after treatment with 100 μM CZ-48 in the absence or presence of 0.4 μM GA. Data: mean ± SD, *n* = 3. Statistics: Student’s *t*-test, *** *p* < 0.001. (**G**) Immunoblot analysis of SARM1 after treatment with 0.4 μM GA, 100 μM CZ-48, or CZ-48 plus GA for the indicated times. Tubulin is shown as loading control.

We previously showed that the cell-permeant NMN mimetic CZ-48 activates SARM1 and induces non-apoptotic cell death in SARM1-overexpressing HEK293 cells [8]. After CZ-48 treatment, cells showed marked shrinkage, prominent blister-like membrane blebbing, and eventual plasma membrane rupture in phase-contrast microscopy and live-cell imaging (Figure 1A and Supplementary Movies S1–S4). These features resemble necroptosis, a regulated necrotic death pathway classically mediated by the RIPK1-RIPK3-MLKL axis [29]. We therefore asked whether CZ-48-induced SARM1 activation engages canonical necroptotic machinery.

We tested pharmacological inhibitors that target different components or regulators of necroptotic signaling: GSK872, a RIPK3 kinase inhibitor [30]; necrosulfonamide (NSA), an inhibitor of MLKL oligomerization [31]; and geldanamycin (GA), an HSP90 ATPase inhibitor that can modulate necroptotic signaling by affecting the stability and function of necroptosis-related kinases such as RIPK1 [32] (Figure 1B). Because GA produced a strong effect in preliminary experiments, we examined it first (Figure 1C). Flow cytometric analysis of PI uptake showed that 100 μM CZ-48 induced a substantial PI-positive population, accounting for approximately 60% of cells. Co-treatment with 0.4 μM GA reduced this population to approximately 14%, indicating protection against CZ-48-induced membrane permeabilization and cell death (Figure 1D).

We then compared the dose-dependent effects of GA, GSK872, and NSA. GA protected cells even at 0.2 μM and substantially reduced CZ-48-induced PI positivity. GSK872 also produced detectable protection, although less completely than GA. NSA showed only limited protection across the tested concentration range (Figure 1E). Thus, CZ-48-induced SARM1-dependent cell death shares some pharmacological sensitivities with necroptotic signaling but does not strictly follow the canonical MLKL-dependent pathway.

A time-course analysis gave the same conclusion. In cells treated with CZ-48 alone, PI-positive cells accumulated over time and reached approximately 60% by 16 h. Co-treatment with GA suppressed PI-positive cells throughout the time course and kept cell death at a much lower level (Figure 1F), indicating that GA substantially inhibits CZ-48-induced SARM1-dependent cell death rather than simply delaying its onset. Because HSP90 inhibition can destabilize client proteins, we asked whether GA protected cells by lowering SARM1 protein abundance. Immunoblotting showed that SARM1 levels remained largely unchanged after treatment with GA, CZ-48, or both over the tested time course (Figure 1G). Thus, GA-mediated protection cannot be explained by SARM1 depletion.

These results show that pharmacological activation of SARM1 by CZ-48 induces a regulated PI-positive death program with necroptosis-like morphology. The inhibitor profile, strong protection by GA, partial protection by GSK872, and minimal protection by NSA argue against a simple assignment to canonical RIPK1-RIPK3-MLKL-dependent necroptosis. The data support a distinct SARM1-driven necrotic death pathway, referred to here as sarmoptosis, that is highly sensitive to geldanamycin but does not require full engagement of canonical necroptotic execution machinery.

### 3.2. VER-155008(VER) and Related ATP-Competitive Compounds Suppress SARM1 Activation-Induced Cell Death and Preserve NAD and ATP

**Figure 2:**
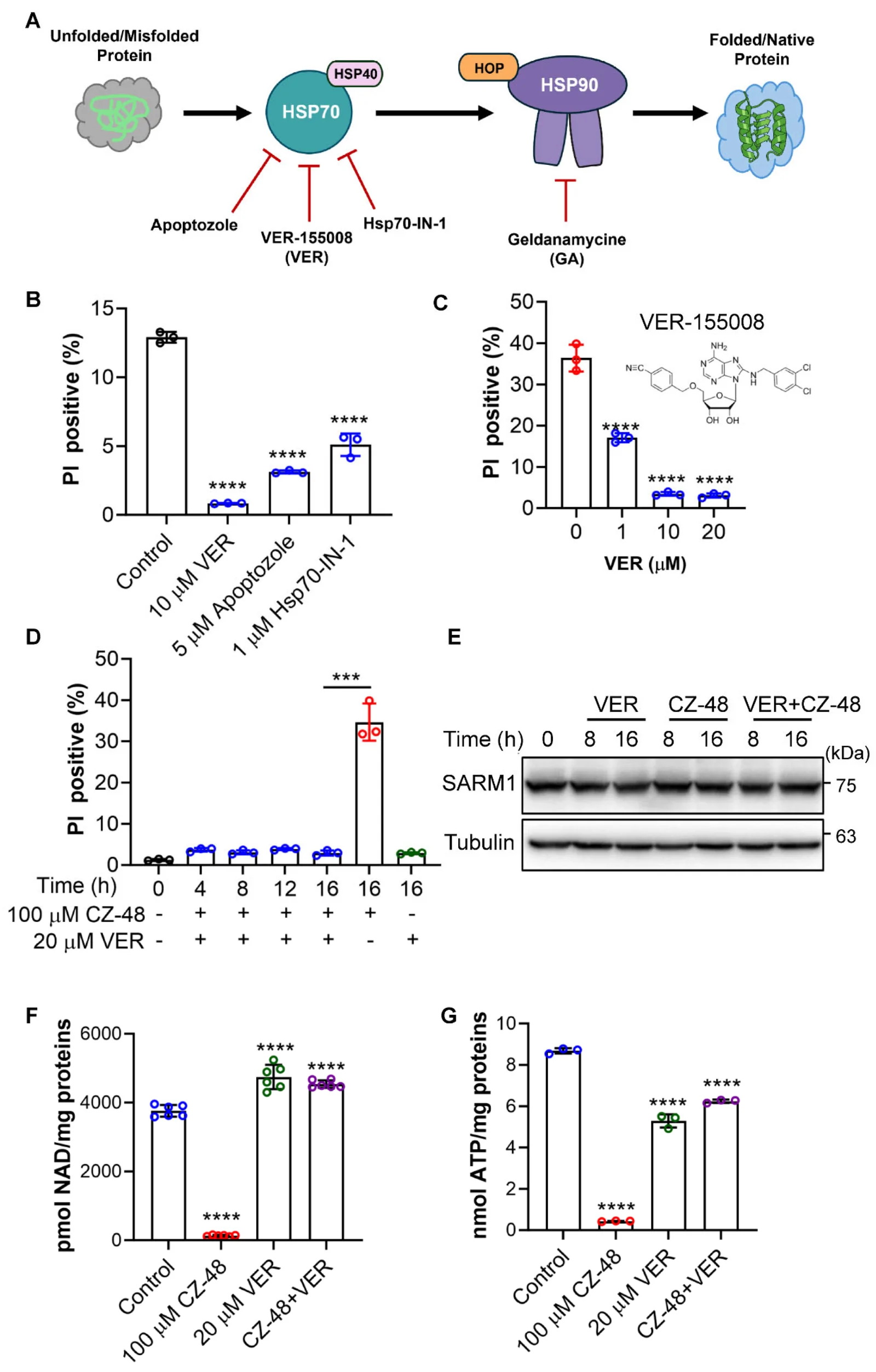
VER-155008 and related HSP70-annotated compounds suppress SARM1 activation-induced cell death. (**A**) Schematic summarizing the chaperone-pathway annotation of VER-155008 (VER), apoptozole, HSP70-IN-1, and GA. (**B**) Quantification of PI-positive cells after treatment with 100 μM CZ-48 for 16 h in the presence of 10 μM VER, 5 μM apoptozole, or 1 μM HSP70-IN-1. (**C**) Dose–response analysis of VER protection against CZ-48-induced PI positivity; the chemical structure of VER is shown. (**D**) Time-course quantification of PI-positive cells after treatment with 100 μM CZ-48 in the absence or presence of 20 μM VER. (**E**) SARM1-OE HEK-293 cells were treated with 20 μM VER, 100 μM CZ-48, or VER plus CZ-48 for the indicated times. Immunoblot analysis of SARM1 was performed using Western blot, with tubulin serving as a loading control. (**F**,**G**) After treatment with the indicated compounds for 16 h, cellular NAD (**F**) and ATP (**G**) measurements were performed with cycling assay or luciferase assay, respectively. Data: mean ± SD, *n* = 3. Statistics: Student’s *t*-test (**D**), one-way ANOVA (**B**,**C**,**F**,**G**); *** *p* < 0.001, **** *p* < 0.0001.

Because GA protected cells from CZ-48-induced death, we asked whether other chaperone-pathway-related compounds had similar effects. We tested compounds annotated or reported as HSP70 inhibitors, including VER [33], apoptozole [34], and HSP70-IN-1 [35] (Figure 2A). All three HSP70-annotated compounds reduced CZ-48-induced PI positivity, with different protective strengths. VER had the strongest effect and reduced the CZ-48-induced PI-positive population to near-baseline levels under the tested conditions (Figure 2B). We therefore focused on VER for subsequent mechanistic work. VER, whose chemical structure is shown in Figure 2C, suppressed CZ-48-induced cell death in a dose-dependent manner. Protection was detectable at 1 μM and was nearly complete at 10–20 μM (Figure 2C). Time-course analysis confirmed the sustained protective effect of VER. CZ-48 alone caused a progressive increase in PI-positive cells, whereas co-treatment with VER almost completely prevented PI uptake at the 16 h endpoint (Figure 2D). These data show that VER potently suppresses CZ-48-induced SARM1-dependent cell death, even more strongly than GA.

To determine whether VER acts by reducing SARM1 protein abundance, we compared cells treated with vehicle, VER alone, CZ-48 alone, or VER plus CZ-48. Immunoblotting showed that SARM1 levels remained largely unchanged across these conditions and time points (Figure 2E). As with GA, VER-mediated protection cannot be attributed to SARM1 protein depletion.

These results identify HSP70-annotated ATP-competitive compounds, especially VER, as suppressors of SARM1 activation-induced cell death. VER protects without reducing SARM1 protein expression and preserves NAD and ATP pools during CZ-48-induced SARM1 activation.

### 3.3. FK866 Separates Generic NAD Depletion from SARM1-Linked ATP Collapse, and Activated SARM1 Reduces ATP In Vitro

**Figure 3:**
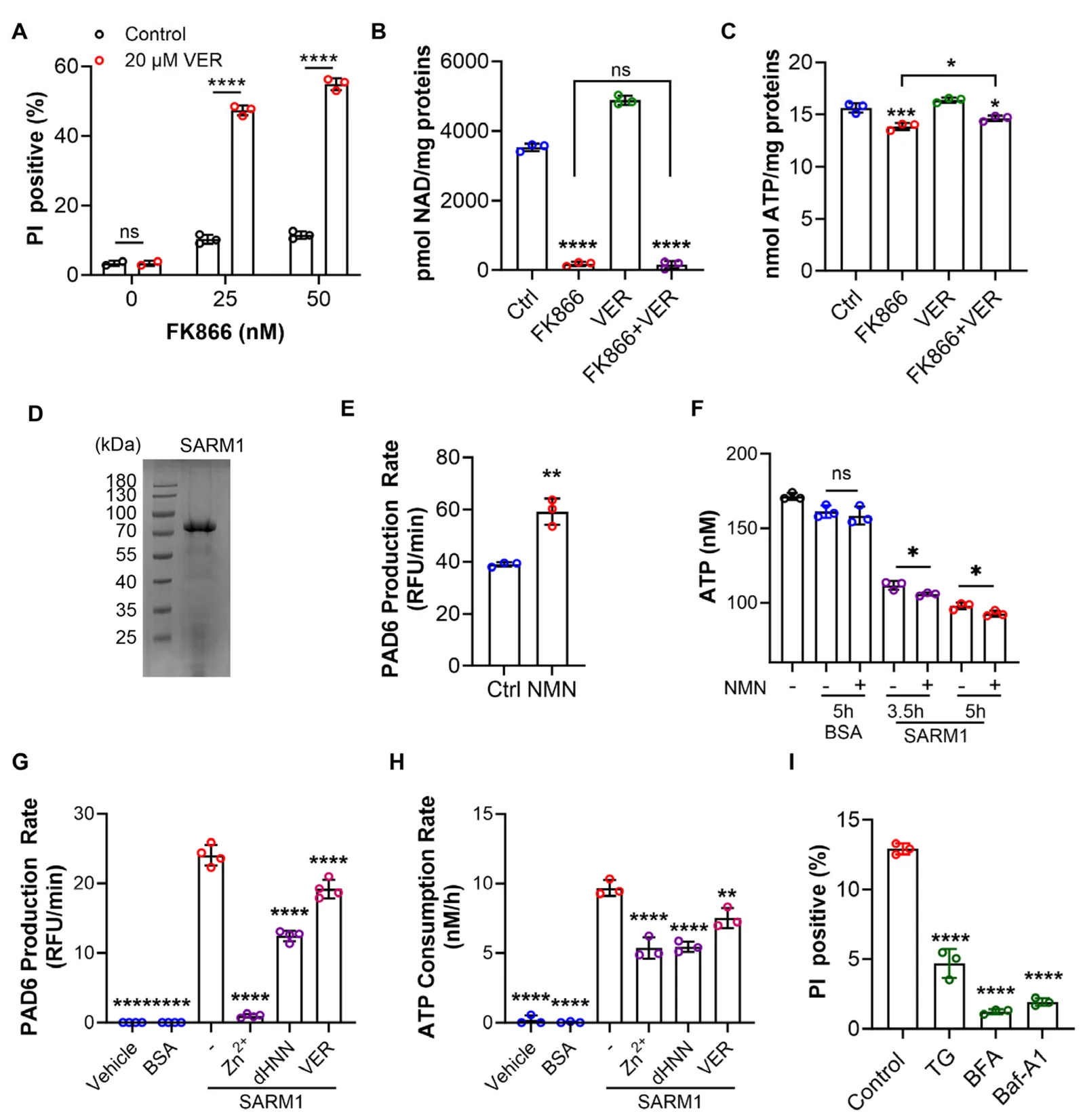
Activated SARM1 directly consumes ATP and supports an ATP collapse model of sarmoptosis. (**A**) Quantification of PI-positive cells after FK866 treatment at the indicated concentrations in the absence or presence of 20 μM VER. (**B**,**C**) NAD (**B**) or ATP (**C**) measurements in cells treated with vehicle, 100 nM FK866, 20 μM VER, or both compounds. (**D**) SDS-PAGE followed by Coomassie Blue staining of purified recombinant SARM1. (**E**) Cell-free PC6 assay showing the base-exchange activity of SARM1 (1 μg/mL) in the presence or absence of 100 μM NMN. (**F**) Cell-free ATP assay showing ATP levels in reactions containing 2 μM recombinant SARM1 with or without 100 μM NMN for 3.5 or 5 h. BSA was included as a negative control. (**G**) Cell-free PC6 assay assessing SARM1 base-exchange activity in the presence of the compounds, including 100 μM Zn^2+^, 100 μM dHNN, and 20 μM VER. BSA was included as a negative control. (**H**) ATPase activity of 2 μM purified SARM1 in the presence of the same compounds as in (**G**). (**I**) Representative PI flow-cytometry histograms of SARM1-overexpressing cells treated with 80 μM CZ-48 alone or together with 0.5 μM bafilomycin A1 (Baf-A1) or 50 nM thapsigargin (TG) for 16 h. For quantified data, data are presented as mean ± SD, *n* = 3. Statistical significance was determined by Student’s *t*-test for panels (**A**,**E**,**F**) and by one-way ANOVA for panels (**G**–**I**). For panels (**B**,**C**), one-way ANOVA followed by Dunnett’s multiple-comparisons test was used to compare the indicated groups with the VER group, and an unpaired two-tailed Student’s *t*-test was additionally used for the planned comparison between FK866 and FK866+VER. ns = not significant, * *p* < 0.05, ** *p* < 0.01, *** *p* < 0.001, **** *p* < 0.0001.

To test whether VER generally protects cells from NAD depletion-induced metabolic stress, we examined its effect in FK866-treated cells. FK866 inhibits NAMPT and blocks NAD salvage biosynthesis [36]. If VER simply blocked downstream consequences of low NAD, it should also protect against FK866. Instead, FK866 induced PI-positive death in a dose-dependent manner, and VER did not rescue this phenotype. Under FK866 conditions, VER further increased the PI-positive population (Figure 3A). VER therefore does not act as a general cytoprotective compound against NAD depletion [36].

Nucleotide measurements supported this boundary. FK866 caused profound NAD depletion, and VER co-treatment failed to restore NAD under FK866-treated conditions (Figure 3B). ATP measurements showed a different kinetic and pharmacological profile from the CZ-48 setting. FK866 produced a slower or more modest ATP decrease at the examined time point, and VER did not restore ATP in FK866-treated cells (Figure 3C). Thus, CZ-48-induced SARM1 activation differs from NAMPT blockade: ATP collapse is faster and VER-sensitive in the SARM1 activation context, but not in the FK866/NAD-salvage blockade context.

These observations prompted us to ask whether SARM1 activation could promote ATP loss through mechanisms beyond NAD depletion. SARM1 contains a TIR enzymatic domain [7,8], and TIR-domain proteins can catalyze diverse nucleotide-related reactions [17,18]. Although SARM1 is best known for its NAD-related activities, the rapid ATP depletion induced by CZ-48 raised the possibility that activated SARM1 might also act on ATP.

To examine this possibility, we prepared purified recombinant SARM1 (Figure 3D) and confirmed its enzymatic activity using the PC6-based NAD base-exchange assay [23]. Recombinant SARM1 showed significant basal PAD6 production activity, indicating that the protein was partially activated during purification, and addition of NMN further increased this activity by approximately 50% (Figure 3E). We then incubated purified SARM1 directly with ATP in a cell-free system. Compared with ATP alone or BSA controls, reactions containing SARM1 showed reduced ATP levels over time, and NMN modestly accelerated this ATP decrease (Figure 3F). Although the activity was relatively weak under these assay conditions, these results support the presence of a SARM1-associated ATP-consuming activity *in vitro*.

We next asked whether this ATP-consuming activity was sensitive to known SARM1 inhibitors. Because many base-exchange inhibitors require NAD-dependent conversion [37,38], we focused on Zn^2+^ and dHNN, which inhibit SARM1 through different mechanisms: Zn^2+^ targets the SS loop in the TIR domain [39], whereas dHNN covalently modifies the ARM domain to prevent SARM1 activation [23]. Both inhibitors reduced SARM1-mediated PAD6 production, with Zn^2+^ showing stronger inhibition than dHNN (Figure 3G). In contrast, Zn^2+^ and dHNN suppressed SARM1-associated ATP consumption to a similar extent (Figure 3H). These results support a link between ATP decrease and SARM1 enzymatic activity, while suggesting that ATP consumption may involve a catalytic state distinct from NAD base-exchange activity. VER also partially inhibited both activities (Figure 3G,H), possibly through ATP competition.

Together, these biochemical data suggest that ATP loss may be a central event in CZ-48-induced sarmoptosis. To further test whether reducing cellular ATP consumption can influence cell death, we treated cells with inhibitors of major housekeeping ATP-consuming pathways. Thapsigargin (TG), which inhibits the SERCA Ca^2+^-ATPase [40], and Bafilomycin A1 (Baf-A1), which inhibits the vacuolar H^+^-ATPase [41], reduced CZ-48-induced PI-positive cell death (Figure 3I). Together, these findings support an ATP-collapse model of sarmoptosis, in which ATP depletion is a central driver of cell death and limiting either SARM1-associated ATP consumption or conventional ATP-consuming processes can alleviate CZ-48-induced cell death.

Given that SARM1-associated ATP consumption was relatively modest in the cell-free assay, we hypothesized that SARM1 activation may engage additional cellular pathways that further promote ATP depletion. We therefore performed iTRAQ-based quantitative proteomic analysis to identify candidate processes, particularly metabolic and mitochondrial alterations, that could contribute to the amplification of ATP loss.

### 3.4. iTRAQ Proteomics and Mitochondrial Staining Identify Mitochondrial Dysfunction as a VER-Rescued Route Linked to ATP Collapse

**Figure 4:**
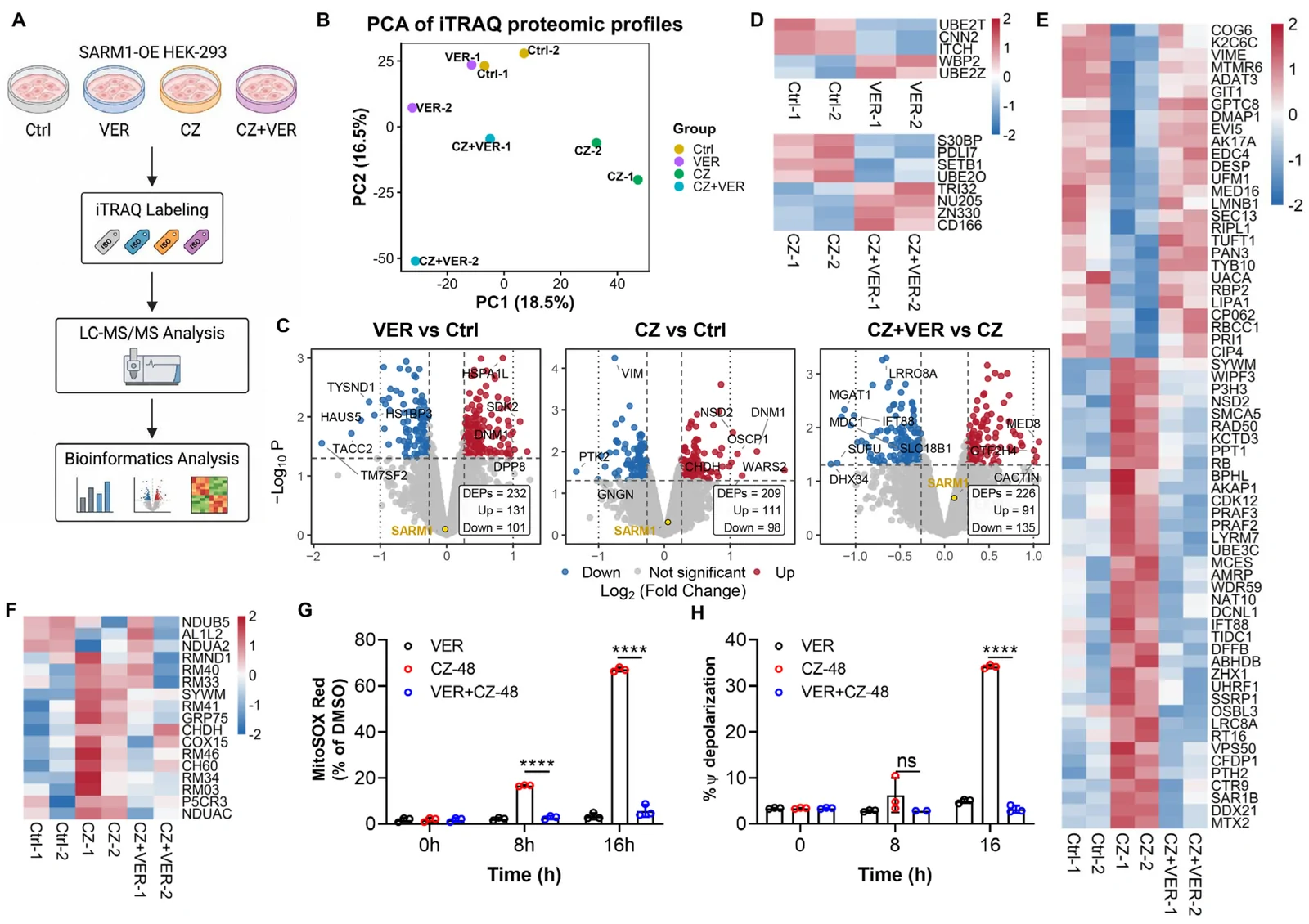
iTRAQ proteomics maps CZ-48- and VER-associated mitochondrial function states. (**A**) Experimental workflow for iTRAQ-based quantitative proteomics in SARM1-overexpressing HEK293 cells treated with vehicle control, 20 μM VER, 100 μM CZ-48, or both compounds. Cells were processed for iTRAQ labeling, LC-MS/MS analysis, and downstream bioinformatic analysis. (**B**) Principal-component analysis of iTRAQ proteomic profiles from the indicated treatment groups. (**C**) Volcano plots showing differentially expressed proteins in VER versus Ctrl, CZ versus Ctrl, and CZ versus CZ+VER comparisons. Upregulated and downregulated proteins are shown in red and blue, respectively; non-significant proteins are shown in gray. Labeled proteins indicate the top changed candidates displayed in the source figure. Differentially expressed proteins (DEPs) were defined using *p* < 0.05 and |FC| ≥ 1.2. (**D**) Heatmap of differentially changed proteins belonging to the predefined HSP70 client protein set in the VER versus Ctrl and CZ+VER versus CZ comparisons. (**E**) Heatmap of CZ-48-responsive, VER-opposed proteins. Proteins shown in this panel met *p* < 0.05 and |FC| ≥ 1.2 in both the CZ-versus-Ctrl and CZ-versus-CZ+VER comparisons and showed either CZ > Ctrl and CZ > CZ+VER or CZ < Ctrl and CZ < CZ+VER. (**F**) Focused heatmap of representative mitochondrial and metabolic proteins. These proteins were selected to represent major mitochondrial functional modules rather than as specific mitochondrial markers. (**G**) MitoSOX Red analysis of mitochondrial superoxide in cells treated with 20 μM VER, 100 μM CZ-48, or VER plus CZ-48 for the indicated times. (**H**) Quantification of mitochondrial depolarization in cells treated as in (**G**), measured by DiOC6(3) staining assay. Data: mean ± SD, *n* = 3. Statistics: Student’s *t*-test (**G**,**H**); ns = not significant, **** *p* < 0.0001.

SARM1-overexpressing HEK293 cells were treated under four conditions: vehicle control, VER, CZ-48, or CZ-48 plus VER, hereafter referred to as Ctrl, VER, CZ, and CZ+VER, respectively. Proteins were extracted, digested, labeled with iTRAQ reagents, and analyzed by LC-MS/MS, followed by group-level comparison and focused bioinformatic analysis (Figure 4A). Principal-component analysis separated the treatment groups, indicating that each condition produced a distinct proteomic state (Figure 4B). Volcano plots summarized protein changes in VER versus Ctrl, CZ-48 versus Ctrl, and CZ-48 versus CZ-48 plus VER comparisons (Figure 4C). The available iTRAQ data contained quantitative abundance measurements for 4318 proteins. Using thresholds of *p* < 0.05 and |log_2_FC| ≥ log_2_(1.2), VER treatment alone altered the abundance of 232 proteins relative to Ctrl, including 131 increased and 101 decreased proteins. CZ-48 treatment significantly affected 209 proteins, comprising 111 increased and 98 decreased proteins, indicating broad but relatively balanced proteomic remodeling. Comparison of CZ with CZ+VER identified 226 differentially abundant proteins, of which 91 were more abundant and 135 less abundant in the CZ group, indicating that a larger subset of proteins increased following VER co-treatment. Collectively, these findings show that VER not only altered the proteome independently but also substantially reconfigured the CZ-48-induced proteomic response.

We first asked whether treatment altered proteins within the predefined HSP70 client protein set. Focused analysis identified five differentially abundant HSP70 client proteins in the VER versus Ctrl comparison and eight in the CZ+VER versus CZ comparison (Figure 4D). In the VER versus Ctrl comparison, UBE2T, CNN2, and ITCH showed decreased abundance following VER treatment, whereas WBP2 and UBE2Z showed increased abundance. In the CZ+VER versus CZ comparison, S30BP, PDLI7, SETB1, and UBE2O showed decreased abundance after VER co-treatment, whereas TRI32, NU205, ZN330, and CD166 showed increased abundance. These findings indicated that VER modulated multiple HSP70 client proteins both when administered alone and in the context of CZ-48 treatment. However, the changes were heterogeneous and did not center on a single dominant HSP70 client. This argues against a model in which VER protects by correcting a single HSP70-client-dependent execution protein.

We next compared protein abundance patterns among the Ctrl, CZ, and CZ+VER groups to identify CZ-48-responsive proteins whose changes were opposed by VER (Figure 4E). Proteins were retained only if they met the criteria of *p* < 0.05 and |FC| ≥ 1.2 in both the CZ versus Ctrl and CZ versus CZ+VER comparisons, and if their abundance in the CZ group was either higher or lower than that in both other groups. Proteins enriched in the Ctrl group, including cytoskeletal and cell junction-associated proteins such as VIME (vimentin), DESP (desmoplakin), LMNB1 (lamin B1), and GIT1, were markedly downregulated following CZ-48 treatment, suggesting that CZ-48 exposure disrupts cytoskeletal organization and structural integrity. In contrast, a distinct cluster of proteins associated with DNA damage response, cell cycle regulation, and ubiquitin-mediated protein degradation, including CDK12, RAD50, UBE3C, DFFB, and SSBP1, showed strong upregulation upon CZ-48 treatment, indicating activation of cellular stress and damage-repair pathways. Notably, this upregulated cluster also included SSBP1, a mitochondrial single-stranded DNA-binding protein essential for mitochondrial DNA replication and maintenance [42], raising the possibility that CZ-48 treatment may induce mitochondrial genomic stress in addition to nuclear damage responses. Together, this CZ-48-responsive, VER-opposed protein set highlights cytoskeletal disruption and activation of stress- and damage-response pathways after CZ-48 treatment, both of which were partially shifted toward the control state by VER co-treatment.

Given that mitochondrial dysfunction and consequent bioenergetic failure could amplify rapid ATP collapse, we next focused specifically on mitochondrial and metabolic proteins to further characterize this response (Figure 4F). CZ-48 altered the abundance of multiple proteins associated with oxidative phosphorylation, mitochondrial translation, redox metabolism, and mitochondrial stress responses. We selected SYWM, P5CR3, NDUB5, NDUAC, NDUA2, COX15, AL1L2, CHDH, GRP75, CH60, RMND1, RM34, RM03, RM33, RM46, RM40, and RM41 as functional representatives of these processes. Many of these changes shifted toward the control group with VER co-treatment. This pattern is consistent with mitochondrial metabolic stress after SARM1 activation and partial reversal by VER.

We then tested whether the proteomic mitochondrial signal was accompanied by functional mitochondrial injury. CZ-48 increased mitochondrial reactive oxygen species, detected by MitoSOX Red staining, whereas VER co-treatment suppressed this increase (Figure 4G). CZ-48 also caused mitochondrial membrane depolarization, measured by DiOC6(3), and VER reduced the depolarized mitochondrial population (Figure 4H). These staining experiments confirmed mitochondrial dysfunction after CZ-48 treatment and functional rescue by VER.

### 3.5. Working Model

**Figure 5:**
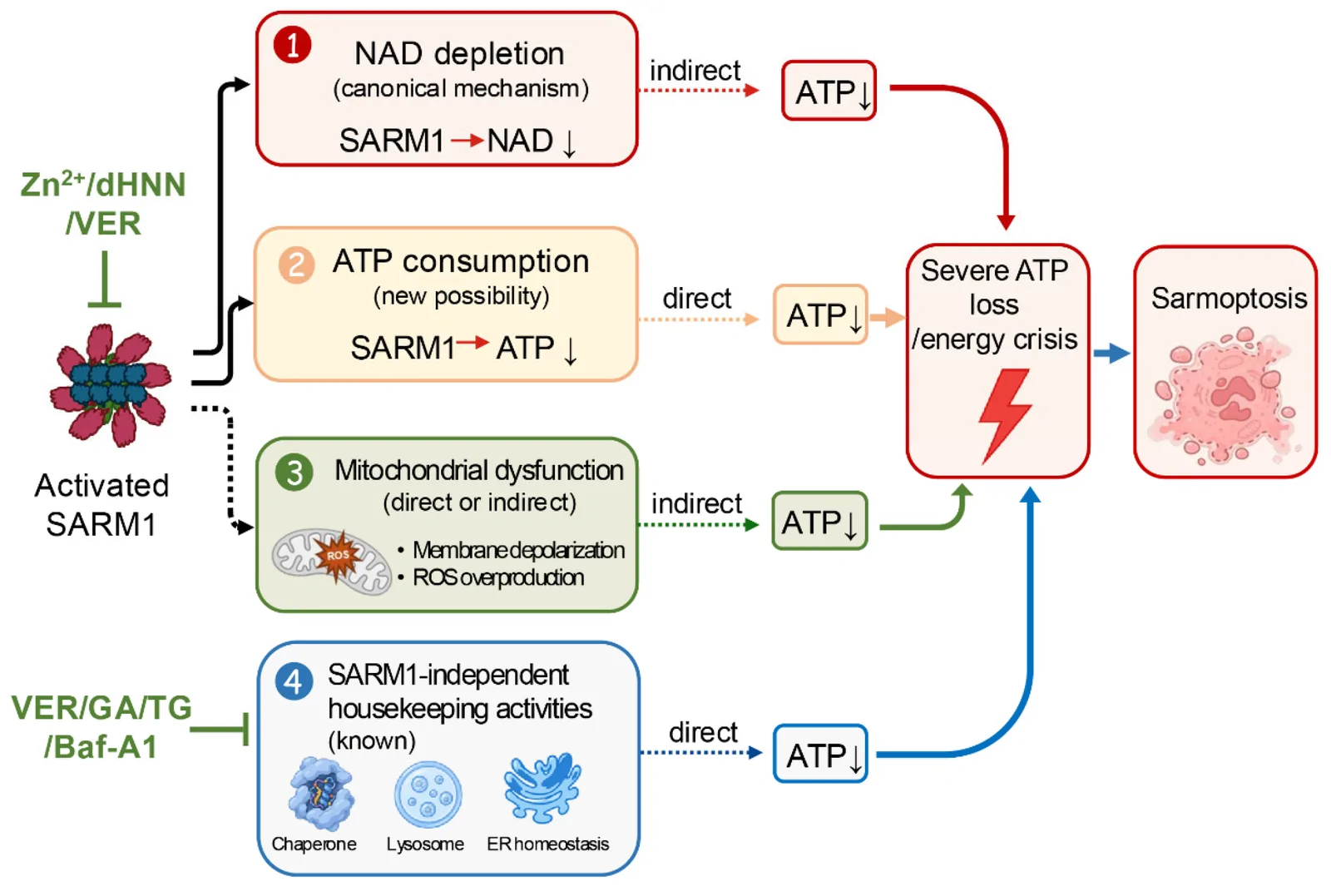
Working model of SARM1-driven ATP collapse and sarmoptosis. This arrow indicates downregulation.

Based on our findings in SARM1-overexpressing HEK293 cells, we propose a revised working model in which CZ-48-activated SARM1 induces sarmoptosis primarily through ATP collapse (Figure 5). This energetic failure may arise through several convergent and non-mutually exclusive mechanisms. First, activated SARM1 impairs NAD-dependent bioenergetic pathways required for ATP production through its canonical NAD-consuming activities. Second, activated SARM1 may directly promote ATP loss through less-characterized enzymatic activities. Third, mitochondrial dysfunction, reflected by increased mitochondrial ROS, DiOC6(3)-detected mitochondrial depolarization, and mitochondrial/metabolic proteomic remodeling, may further restrict ATP-generating capacity. VER protects against sarmoptosis by blocking these SARM1-dependent events, thereby preserving NAD-dependent ATP homeostasis, limiting ATP loss, and mitigating mitochondrial injury. Moreover, its protective effect may also involve suppression of SARM1-independent ATP-consuming pathways, in line with the rescue observed with ATPase-targeting compounds such as GA, TG and Baf-A1. Collectively, these results indicate that ATP collapse is a central driver of sarmoptosis and that restoring ATP balance, either by sustaining ATP production or by reducing ATP consumption, is sufficient to attenuate cell death in this CZ-48/SARM1-dependent model.

## 4. Discussion

The main finding of this study is that SARM1 activation produces a rapid, pharmacologically modifiable ATP-collapse phenotype that cannot be fully explained by NAD depletion alone. In CZ-48-treated SARM1-overexpressing HEK293 cells, SARM1 activation induced regulated PI-positive death, NAD and ATP depletion, and necrosis-like morphology. GA and VER strongly reduced death without lowering SARM1 abundance, while VER preserved NAD and ATP only in the CZ-48/SARM1 setting and not during FK866-mediated NAD starvation. Purified SARM1 reduced ATP levels *in vitro*, and the iTRAQ, MitoSOX Red, and DiOC6(3) data further linked CZ-48 treatment to mitochondrial/metabolic remodeling, mitochondrial ROS, and mitochondrial depolarization that were mitigated by VER. Together, these results support a multi-route ATP-collapse model rather than a single-pathway explanation.

This model refines, but does not replace, the established view of SARM1 as an inducible NADase in axon degeneration. Prior studies showed that SARM1 activation destroys NAD, placing NAD loss upstream of axonal metabolic failure. Our FK866 comparison is consistent with that framework but shows that generic NAMPT-dependent NAD starvation differs from CZ-48-induced SARM1 activation in kinetics and VER sensitivity. The cell-free ATP result is also consistent with the broader concept that TIR domains can catalyze diverse nucleotide reactions [17,18]. However, this finding should be interpreted cautiously. First, the ATP-consuming activity detected *in vitro* was modest, suggesting that the current assay conditions may not fully recapitulate the optimal molecular environment, conformational state, cofactor requirements, or downstream amplification mechanisms present in cells. Second, the reaction products remain to be characterized; therefore, ATP disappearance alone does not define whether SARM1 directly hydrolyzes ATP or converts ATP into other nucleotide-derived products that may further amplify ATP depletion in cells. Third, trace ATPase contamination in the recombinant protein preparation cannot be completely excluded, although the NMN dependence and sensitivity to SARM1 inhibitors support a SARM1-associated ATP-consuming activity. Further biochemical studies using optimized reaction conditions and LC-MS/MS-based product identification will be required to define the precise nature of this reaction.

An additional limitation of this study is the use of SARM1-overexpressing HEK293 cells as the cellular model. Although this system enables robust and synchronized SARM1 activation for mechanistic analysis, the ectopically expressed SARM1 level was not quantitatively calibrated against endogenous neuronal SARM1 levels. Therefore, the kinetics and magnitude of CZ-48-induced ATP depletion observed in this model should be interpreted as reflecting acute SARM1 activation in a sensitized cellular system, rather than the physiological rate of ATP loss in neurons. Thus, whether a comparable ATP-collapse response occurs in neurons with endogenous SARM1 expression remains to be established and may depend on cellular context, activation conditions, and SARM1 expression level.

The broader implication of our work is that sarmoptosis may result from an imbalance among ATP production, ATP consumption, and SARM1-dependent nucleotide metabolism. This model helps explain why ATP-competitive or ATPase-associated compounds can protect cells despite not being SARM1-specific inhibitors. GA, TG, and Baf-A1 likely alleviate ATP crisis mainly by inhibiting conventional ATP-consuming housekeeping processes, including chaperone activity, ER Ca^2+^ handling, and lysosomal acidification. VER may have a dual effect: reducing cellular ATP consumption through inhibition of ATP-dependent chaperone activity, and partially suppressing SARM1 activities.

The iTRAQ analysis supports the idea that CZ-48-induced sarmoptosis involves broad cellular remodeling rather than a single downstream effector. Focused analysis of the predefined HSP70 client protein set identified multiple HSP70 client proteins whose abundance was altered by VER alone or by VER co-treatment in the presence of CZ-48, but the changes were heterogeneous and did not identify a single dominant client protein. More broadly, the proteomic analysis identified multiple proteins altered after CZ-48 stimulation, with several showing directional shifts toward a more control-like abundance pattern following VER co-treatment, consistent with modulation of the CZ-48-induced proteomic response. However, we did not identify a single key protein responsible for ATP depletion; knockdown or overexpression of several candidate genes failed to reproduce VER-mediated protection in preliminary experiments. These results suggest that CZ-48-activated SARM1 may trigger broader remodeling of cellular metabolic status, proteostasis, and organelle homeostasis. In addition, we cannot exclude the possibility that CZ-48-induced activation of overexpressed SARM1 causes enzyme-independent conformational or assembly changes that disrupt mitochondrial organization and ATP production. Nevertheless, because oligomycin, mitochondrial uncoupler, and oxygen-consumption assays were not performed in this study, our current data do not establish mitochondrial respiratory failure as the primary cause of ATP collapse. We therefore interpret the observed mitochondrial changes as associated with, and potentially amplifying, SARM1-linked ATP loss. Future studies using mitochondrial inhibitors, uncouplers, Seahorse-based respiration assays, and earlier time-course analyses will be required to define the causal contribution of mitochondrial ATP-production failure.

Several preclinical studies have shown that pharmacological SARM1 inhibition can protect axons in injury- or chemotherapy-induced neuropathy models [43,44,45], supporting the feasibility of targeting SARM1-dependent degeneration. However, recent reports that some inhibitor classes may cause paradoxical activation or show safety liabilities emphasize that future SARM1 drug development will require careful optimization of potency, selectivity, mechanism of action, and dosing window [46,47,48]. In this context, our study suggests that effective intervention in sarmoptosis may require not only blocking canonical SARM1 NADase activity, but also preserving cellular ATP homeostasis by limiting SARM1-associated nucleotide consumption and mitochondrial dysfunction. Thus, defining how SARM1 activation converges on ATP collapse may provide a useful framework for developing safer and more effective strategies to prevent SARM1-driven cell death.

From the perspective of cell-death biology, sarmoptosis emphasizes that collapse of NAD and ATP metabolism can function as a primary execution mechanism of regulated non-apoptotic cell death. This distinguishes it from caspase-, MLKL-, or gasdermin-driven death pathways and provides a framework linking SARM1 activation, mitochondrial dysfunction, metabolic failure, and loss of cell viability. Therefore, sarmoptosis expands the conceptual landscape of regulated cell death and highlights cellular energy homeostasis as a therapeutic target.

## 5. Conclusions

CZ-48-induced SARM1 activation drives a regulated PI-positive death program best explained by convergent ATP collapse rather than by canonical apoptosis, canonical necroptosis, generic NAD starvation, or a single HSP70-centered pathway. VER and related ATP-competitive or ATPase-sensitive interventions protect cells without reducing SARM1 abundance, FK866 separates this protection from general NAD depletion, and purified SARM1 shows modest but convincing ATP-consuming activity. Combined with iTRAQ and mitochondrial staining data, these findings support a working model in which NAD depletion, direct ATP consumption, and associated mitochondrial dysfunction cooperate to produce sarmoptosis.

## Data Availability

The MS proteomics data has been deposited to the ProteomeXchange Consortium via the iProX partner repository, and the project accession code is PXD082054.

## Supplementary Materials

The following supporting information can be downloaded at: https://www.mdpi.com/article/10.3390/biology15171552/s1, Supplementary Movies S1–S4. Recording of cell death in different conditions. Supplementary Table S1. Plasmids, antibodies, and chemical reagents. Supplementary Table S2. Protein expression table from the iTraq analysis, with labels 113 and 114 representing duplicate control samples, labels 115 and 116 representing VER-treated samples, labels 117 and 118 representing CZ-48-treated samples, and labels 119 and 121 representing VER and CZ-48 co-treated samples. Supplementary Table S3. Protein subsets used in the study. Supplementary File S1: Original Western blot images for Figure 1 and Figure 2.
