# Supplementary material for: Beyond NAD Depletion: SARM1-Induced ATP Collapse Involves Direct ATP Degradation and Mitochondrial Dysfunction and Is Pharmacologically Reversible
Source: Biology (Basel). 2026 Sep 4;15(17):1552. doi: 10.3390/biology15171552 (PMC13565343; doi:10.3390/biology15171552)
Supplement: Supplementary file 1 [file biology-15-01552-s001.zip › File S1.pdf]

Fig 1.A

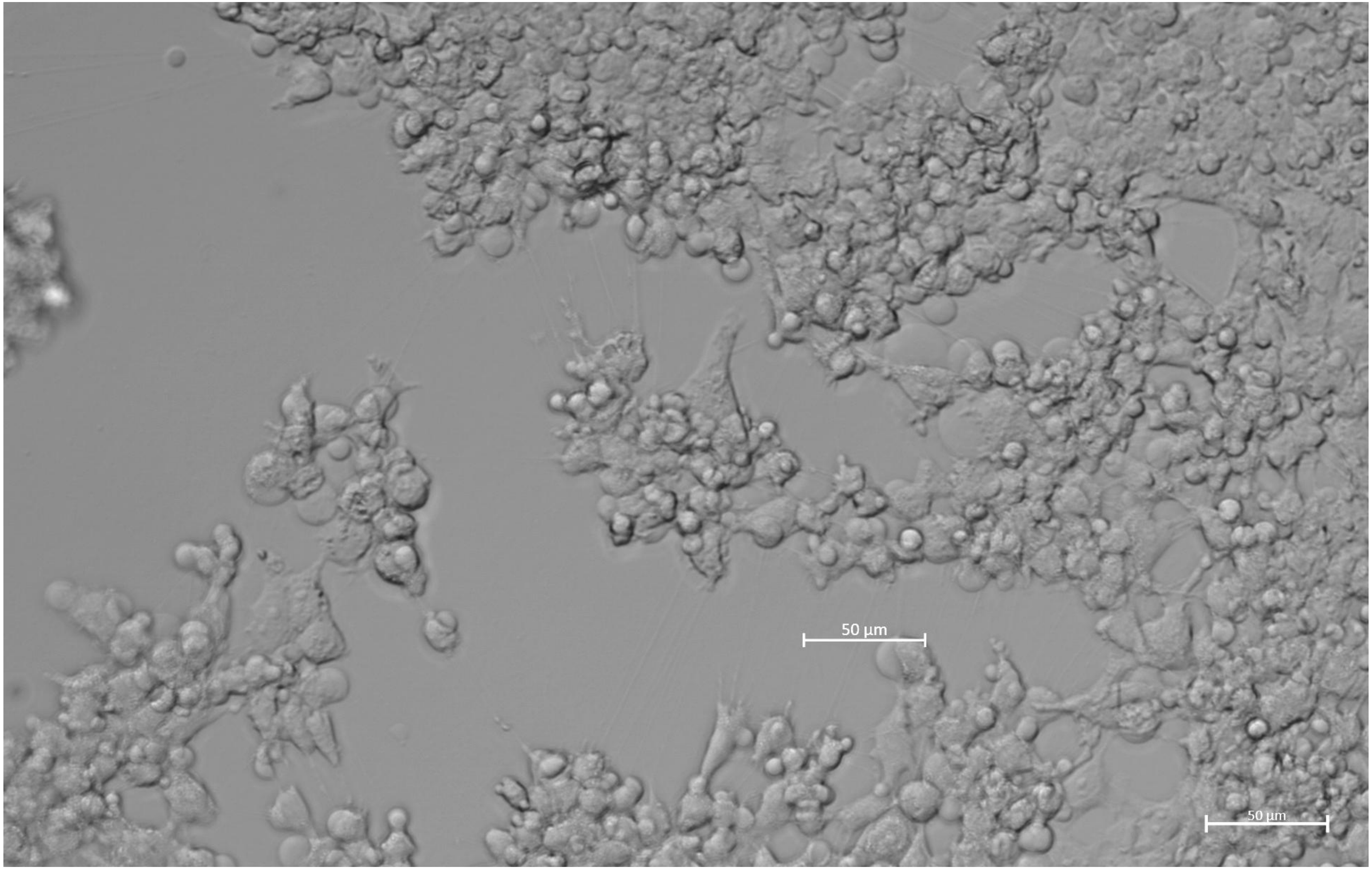

Fig 1.G

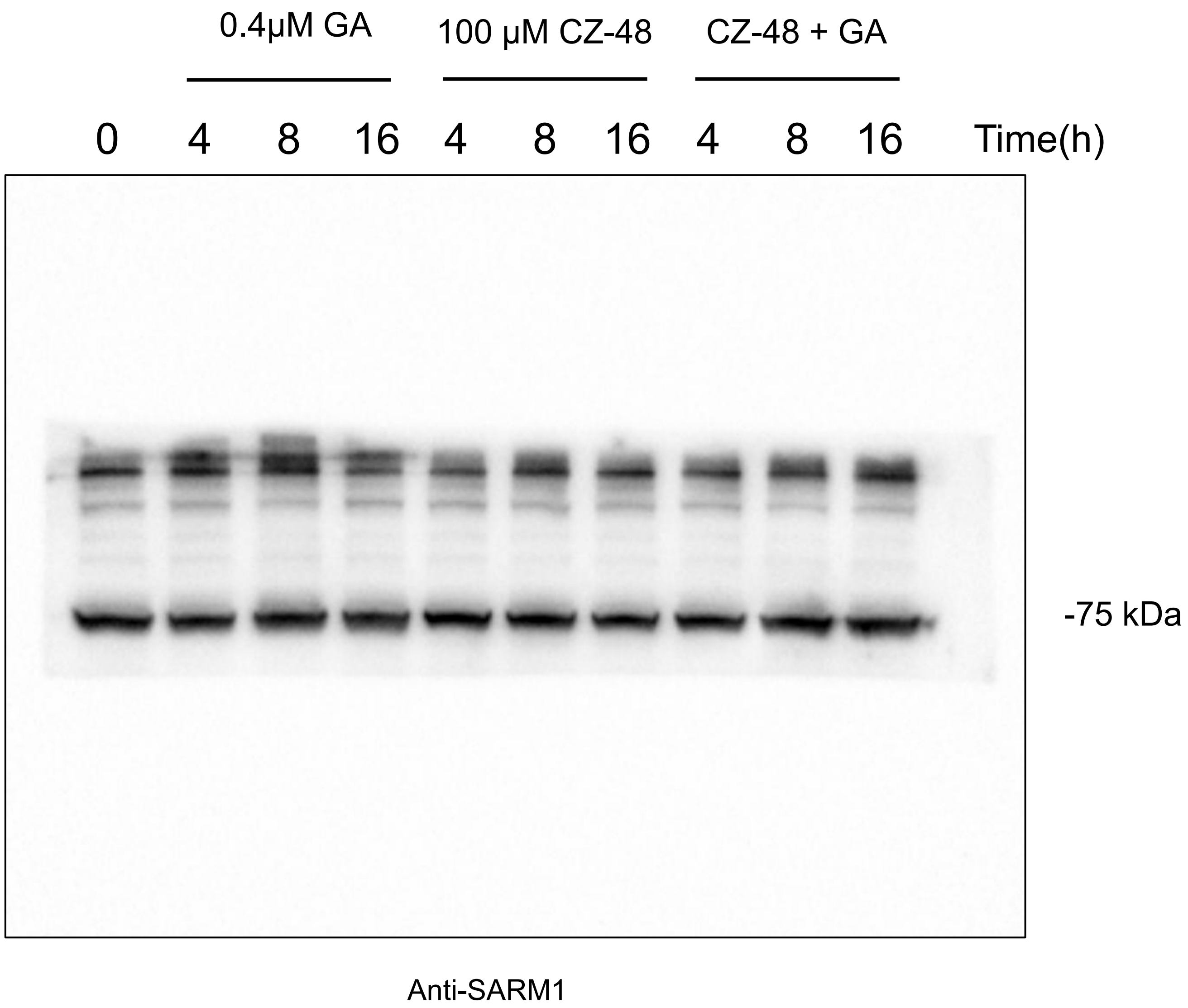

0.4μM GA
100 μM CZ-48
CZ-48 + GA
0 4 8 16 4 8 16 4 8 16 Time(h)
-75 kDa
Anti-SARM1

Fig 1.G

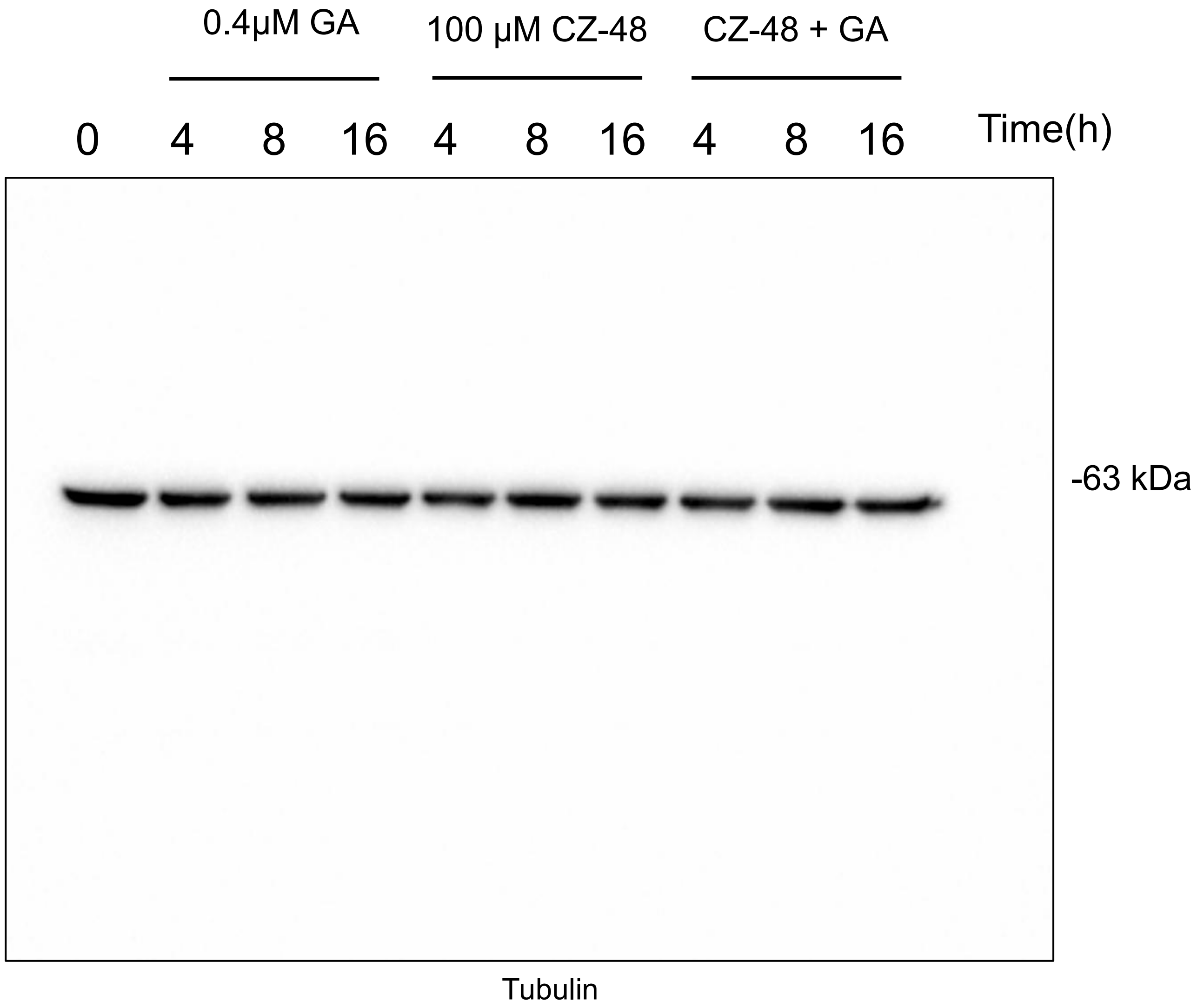
0.4μM GA
100 μM CZ-48
CZ-48 + GA
0 4 8 16 4 8 16 4 8 16
Time(h)
-63 kDa
Tubulin

Fig 2.E

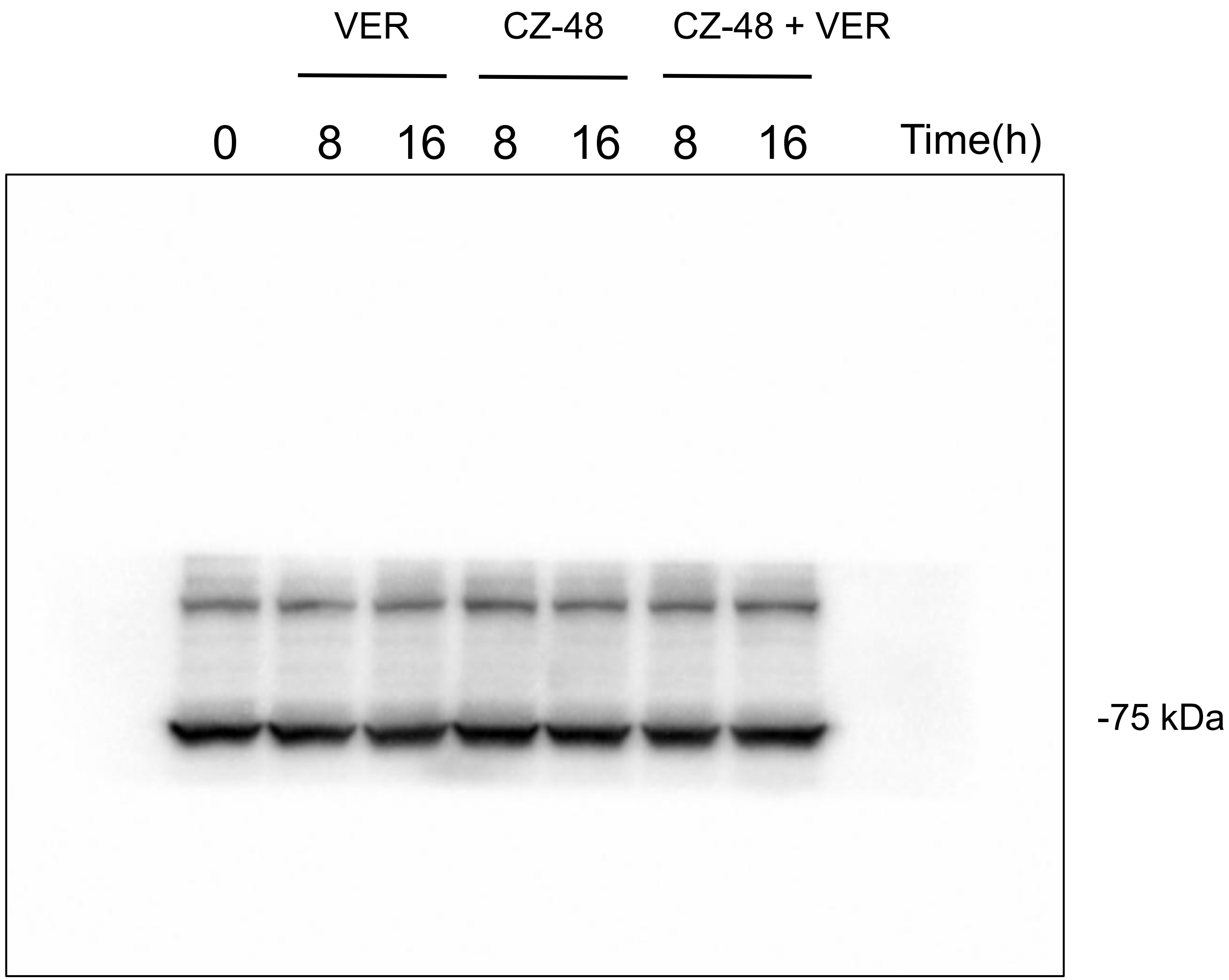

Anti-SARM1

Fig 2.E

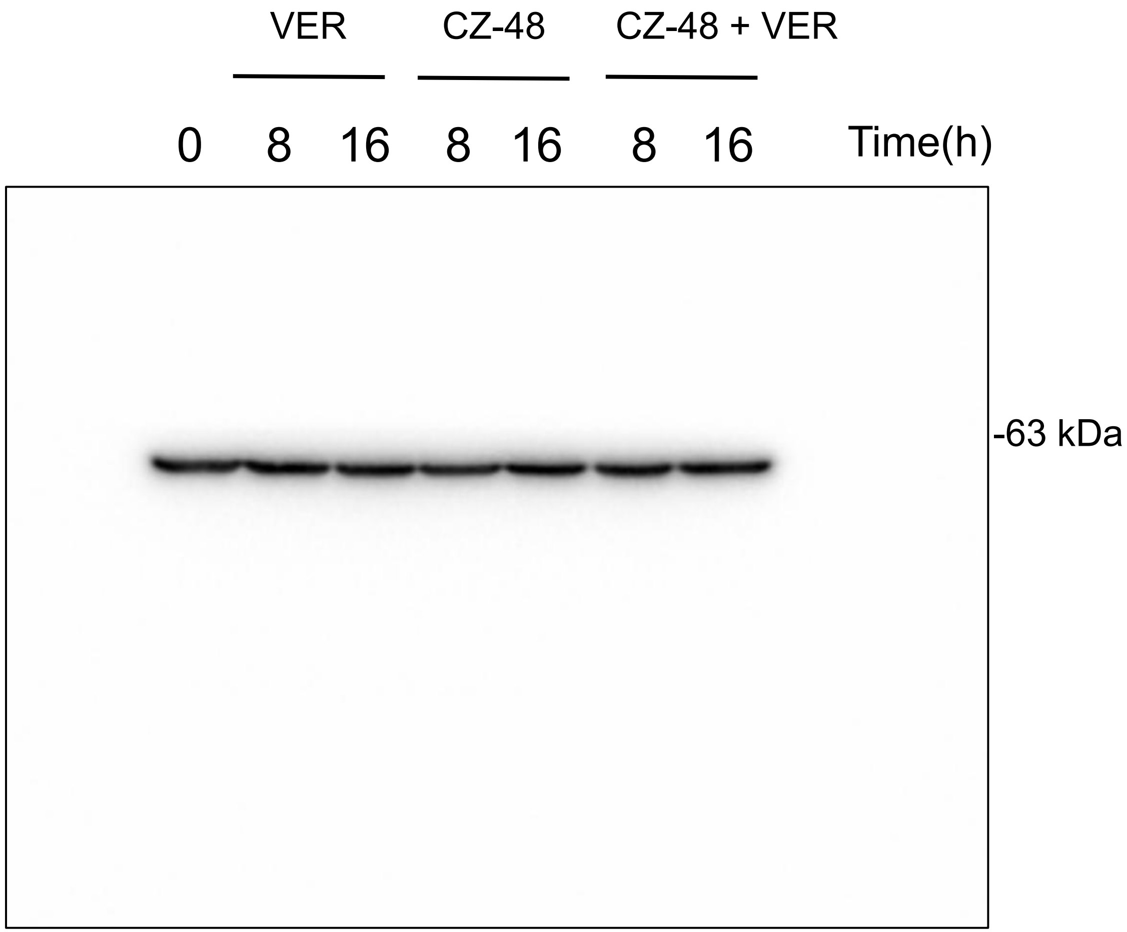

Tubulin
